# Paucity of intellectual property rights information in the US biologics system a decade after passage of the Biosimilars Act

**DOI:** 10.1371/journal.pmed.1004381

**Published:** 2024-04-25

**Authors:** Robin Feldman

**Affiliations:** University of California Law, San Francisco, California, United States of America

## Abstract

In this Policy Forum piece, Robin Feldman discusses how current legislation contributes to informational deficits around drug patents for biologic drugs in the United States.

- The Food and Drug Administration (FDA) maintains 2 lists of drugs approved in the United States—the Orange Book for small-molecule drugs and the Purple Book for large-molecule drugs.
- US law requires that each application for approval of a small-molecule drug disclose the patents protecting the drug and mandates publication of the information in the Orange Book.
- US law, however, imposes no patent-disclosure requirement on any application for approval of a large-molecule drug. Instead, US law requires that a brand biologic company disclose its patents—and that those patents are published in the Purple Book—only *after* a biosimilar applies for approval, and even then, only if rights negotiations between the brand and biosimilar reach a certain stage.
- This legal regime for large-molecule drugs has led to remarkably little patent disclosure in the Purple Book: Only approximately 2% of the brand biologic listings in the Purple Book contain patent information. In comparison, the percentage of small-molecule drugs disclosing patent information in the Orange Book is 21 times greater.
- Given that the Purple Book lists so little patent information about brand biologic drugs, a prospective biosimilar maker cannot readily assess its risk of patent infringement litigation, and so must make investment and manufacturing decisions blindly.
- The informational deficit in the Purple Book contributes to the high price of biologic drugs by discouraging biosimilar entry and hampering competition: Based on the most recent data available, biologics accounted for 37% of all drug spending in the United States while accounting for only 2% of all US prescriptions.

The FDA publishes 2 lists of approved drugs. The lists are known popularly as the “Purple Book” and the “Orange Book.” The Purple Book lists biological drugs, also known as “biologics” or “large-molecule drugs,” while the Orange Book lists small-molecule drugs. Both books are also supposed to identify the patents protecting each listed drug, so that prospective manufacturers of less-expensive copies of the drugs—called “biosimilars” for large-molecule drugs and “generics” for small-molecule drugs—can assess their infringement risk and prepare for entry. In practice, however, the Purple Book provides far less disclosure about patents than the Orange Book. Without knowing what patents are looming, the prospective biosimilar manufacturer must make investment and manufacturing decisions in the dark. That darkness discourages biosimilar entry, reduces competition, and leaves patients and insurers to pay for brand drugs at unnecessarily high prices. Such a result is ironic because price *reduction* was a key goal of the Biologics Price Competition and Innovation Act—also known as the “Biosimilars Act” [1]. Congress passed the Act in 2010 to create an abbreviated pathway for biosimilar entry, just as the Hatch-Waxman Act in 1984 created an abbreviated pathway for generic entry [2].

Although there is a growing recognition that the Purple Book has informational gaps [3], this is the first article to give a comprehensive quantitative assessment of those gaps. As described in detail below, no patents are disclosed at all for the vast majority of drugs listed in the Purple Book.

One prefatory point: The Biosimilars Act created a subcategory of biosimilars named “interchangeables” that must meet heightened approval requirements. Without directly contacting a physician, pharmacists may fill a patient’s biologic prescription using the less-expensive interchangeable, state law permitting. Thus, the data below for biosimilars include the subcategory of interchangeables.

As of November 2023, the Purple Book contains 1,966 entries [4]. Counting entries with the same Biologics License Application (BLA) number as a single entry yields 732 unique BLA numbers [4]. Excluding all BLAs for over-the-counter drugs, discontinued drugs, and biosimilars (including interchangeables) yields 563 unique BLAs for prescription brand biologics [4]. But of those 563 listings, only 11 disclose patent information [4–6]. Those 11 correspond to the following 10 biologic drugs (as some biologic drugs have more than one BLA number): Actemra (tocilizumab), Avastin (bevacizumab), Eylea (aflibercept), Herceptin (trastuzumab), Humira (adalimumab), Lucentis (ranibizumab), Neulasta (pegfilgrastim), Prolia and Xgeva (2 brand names for denosumab), Stelara (ustekinumab), and Tysabri (natalizumab) [6]. (Readers should note that the November 2023 version of the Purple Book erroneously attributed 5 Eylea patents (US patents 11505593, 11548932, 11555176, 11577025, 11559564) to 4 BLAs belonging to other biologics. The FDA corrected its error in the January 2024 edition of the Purple Book. This study, while using the November 2023 edition of the Purple Book, corrects those data by removing the 5 Eylea patents that the FDA erroneously attributed to other biologics at the time, which then yields the correct number of BLAs, 11.)

In short, only approximately 2% of the Purple Book’s unique BLA listings for prescription brand biologics identify at least 1 patent. The paucity of listings identifying at least 1 patent cannot be attributed solely to patent expiration for the following reason. On average, when a drug obtains FDA approval, 12 years of patent protection remain on the key patent [7,8]. Of the 563 unique BLAs in the Purple Book, 275 (48.9%) were approved in or after the year 2012 [4]. Thus, one might have expected that at least some of the 275 drugs would have unexpired patents. Nevertheless, only one of the 275 unique BLAs listed a patent in the Purple Book [4].

Patent disclosure in the Orange Book is significantly more robust. The Orange Book lists New Drug Applications (NDAs) and Abbreviated New Drug Applications (ANDAs). Generally, makers of brand small-molecule drugs file NDAs, while makers of generic small-molecule drugs file ANDAs. Every NDA must identify the patents protecting the drug at issue, and the Orange Book publishes that patent information. Examination of the November 2023 edition of the Orange Book shows that the number of unique prescription brand NDAs was 2,423. Of those, the number listing at least 1 patent was 1,031 [9]. (These figures were obtained by selecting unique NDA numbers and excluding over-the-counter and discontinued drugs. By definition, the figures also exclude ANDAs, i.e., generic drug applications.) That is, the percentage of unique prescription brand NDAs that list at least 1 patent was 42.6% (i.e., 1,031/2,423). Thus, the percentage of patent disclosure is 21 times greater in the Orange Book than in the Purple Book (42.6% compared to 2%).

The relisting of certain drugs provides another basis for an orange-to-purple comparison. Several prescription drugs previously listed in the Orange Book are now listed in the Purple Book [6]. More specifically, in March 2020, the FDA followed a congressional mandate and transferred certain drugs that had been approved under NDAs, reclassifying those NDAs as BLAs, while preserving their application numbers [10]. That transfer included insulins, which are widely prescribed, life-saving drugs. The relevant drugs were accordingly transferred from the Orange Book to the Purple Book and became subject to the Purple Book’s less-demanding requirements for patent disclosure [11]. By comparing NDA numbers from the October 2019 edition of the Orange Book—the last edition published before the transfer—to BLA numbers listed in the November 2023 version of the Purple Book, we found 57 drugs that were moved, including human growth hormone, chorionic gonadotropin, and insulin. When those 57 drugs were listed in the Orange Book, 29 (50.9%) of the Orange Book’s listings for those drugs disclosed patent information [9]. But after those 57 drugs were moved to the Purple Book, not one of the Purple Book’s listings for those exact same drugs disclosed patent information [4–6].

As before, this dearth of patent disclosure in the Purple Book cannot be due to patent expiration. Those 57 drugs, when listed in the Orange Book, identified a total of 325 patents, of which 72 patents expired before November 30, 2023, and 58 patents expired between October 31, 2019 (the approximate date of the Orange Book edition used in this study), and November 30, 2023 (the approximate date of the Purple Book edition used in this study) [9]. Thus, the vast majority of those 325 patents (77.8% or 82.2%, depending on which expiration metric, 72 or 58, is used) were unexpired as of the publication of the November 2023 edition of the Purple Book used in this study.

An additional concern is the absence of patent information for FDA-licensed biologic drugs that have FDA-licensed biosimilars (including interchangeables) and that thus may have gone through some or all of the so-called “patent dance.” The “patent dance” is the carefully choreographed exchange of patent-related information, between the brand biologic and the biosimilar, that was enacted in 2010 as part of the Biosimilars Act [1]. Although complex, the “patent dance” has the following key steps: The biosimilar provides its application and manufacturing process information to the brand; the brand and the biosimilar exchange initial lists of patents arguably infringed by the biosimilar; the brand and biosimilar negotiate a single list of patents arguably infringed by the biosimilar (and, failing an agreement, the parties exchange separate lists of such patents); the brand begins the first phase of litigation by suing the biosimilar for infringement of the patents on the negotiated or separate lists; the biosimilar notifies the brand, 180 days in advance, that the biosimilar will be commercially marketed; and the brand begins the second phase of litigation, in which it can seek a preliminary injunction against the commercial marketing of the biosimilar and which also addresses any patents on the initial lists that were not on the negotiated or separate lists [12]. Subsequent legislation mandated the brand’s initial patent list to be published by the FDA in the Purple Book [13]. By mandating the “patent dance” and publication of the brand’s initial patent list, Congress intended public identification of the patents protecting each brand biologic. The purpose was to enable subsequent biosimilar manufacturers to assess their risk of infringement litigation and to prepare for entry. Congress, however, gave the brand biologic makers significant discretion over how many of their patents to include in the list published by the FDA in the Purple Book [11]. In part because of this discretion, the “patent dance” has led to far less patent disclosure in the Purple Book than is desirable [11,14].

This lack of disclosure is evident in the low number of Purple Book listings that identify any patents at all. In the US, 13 biologic drugs have FDA-licensed biosimilars (including interchangeables) [5,15]. (The biologic drug Enbrel (etanercept) had biosimilars at one point, but those biosimilars have been discontinued.) The 13 biologic drugs are Actemra (tocilizumab), Avastin (bevacizumab), Epogen/Procrit (epoetin alfa), Herceptin (trastuzumab), Humira (adalimumab), Lantus (insulin glargine), Lucentis (ranibizumab), Neulasta (pegfilgrastim), Neupogen (filgrastim), Remicade (infliximab), Rituxan (rituximab), Tysabri (natalizumab), and Stelara (ustekinumab). These correspond to 15 unique BLAs (Rituxan and Stelara each having 2 BLA numbers). The total number of the FDA-licensed biosimilars (including interchangeables) is 45 [15]. The foregoing figures are available as of December 2023, from the Purple Book’s searchable online portal [5,15]. Although the makers of these 13 drugs may have engaged in the “patent dance,” the Purple Book discloses patents for only 8 of the 13: Actemra (tocilizumab), Avastin (bevacizumab), Herceptin (trastuzumab), Humira (adalimumab), Lucentis (ranibizumab), Neulasta (pegfilgrastim), Tysabri (natalizumab), and Stelara (ustekinumab) [6].

Even where the Purple Book discloses patent information for a particular drug, the disclosure is not necessarily exhaustive [16]. For example, although recent reporting reveals that AbbVie, which makes Humira, has obtained 165 patents for the drug [17], the initial patent list it produced during AbbVie’s “patent dance” with Alvotech included only 63 patents [18]. As of November 2023, the Purple Book lists 66 patents for Humira [6]—meaning that almost 100 of Humira’s patents are not disclosed in the Purple Book. The Purple Book’s non-disclosure of patent information cannot be explained by legal exclusion of any patent category: While federal law bars NDAs, and hence the Orange Book, from disclosing process patents and patents on packaging, metabolites, and intermediates [19], federal law imposes no analogous restriction on patent disclosure in the Purple Book, meaning that the number of patents disclosed in the Purple Book should be greater.

There are at least 2 reasons why a brand biologic maker might choose not to disclose all of its patents in the “patent dance,” and might thereby limit disclosure of its patents in the Purple Book, both of which operate to deter biosimilar entry into the market. First, the brand biologic maker might opt to keep some potentially assertable patents in reserve for future biosimilar litigations [16]. That strategic calculation could be risky, however, because of a rarely litigated statutory clause known as the “list it or lose it provision” [20]. The provision’s plain language would prevent the brand biologic from suing under the statute authorizing patent infringement claims—for infringement of any patents that should have been, but were not, included in the brand’s initial patent list provided during the “patent dance” [20].

The second reason resides at the intersection of statutory language and biological complexity. As noted above, the brand’s initial patent list provided during the “patent dance” identifies the patents arguably infringed by the biosimilar [21]. But biological molecules are uniquely complex; even a minute variation in the manufacturing process of a biologic drug can cause a variation in the drug’s molecular structure—for example, its glycosylation profile [22,23]. For this reason, no biosimilar is identical either to its reference brand biologic [23,24] or to a subsequent biosimilar [23]. Thus, the patents that the brand biologic maker thinks are infringed by the first biosimilar may be different from the patents it thinks are infringed by the second biosimilar [16]. The brand biologic maker’s initial patent list may end up disclosing a particular patent to the first biosimilar but not the second, and vice versa. In short, not every patent protecting the brand biologic drug will end up being disclosed in the “patent dance” between the brand biologic maker and the first biosimilar [16].

Since US patents are all publicly available, one might expect the biosimilar makers to be able to find the patents protecting the relevant biologic notwithstanding the Purple Book’s lack of patent disclosure. In reality, however, such a search would be akin to looking for a needle in a haystack, without any idea of what the needle looks like or whether it actually exists. Indeed, patents protecting a drug may not even mention the name of the drug or its active ingredient. A recent study of insulin products found that the last-to-expire patents on almost a third of the products studied did not mention the active ingredient (insulin) at all. Yet, these patents extended the market monopoly of the corresponding insulin products by a median of 4.3 years, delaying biosimilar entries and protecting the brand products’ monopoly pricing [25].

Other possible causal factors for the high price of biologic drugs could include, for example, if rapid expansion of demand for biologics occurred at a time when unexpired protections blocked competitive entry. Without discounting such factors, the high price of biologics is likely due, at least in part, to the Purple Book’s informational deficits. The consequent harm to patients in the aggregate is hardly trivial. Based on the most recent data available [26], biologics accounted for 37% of all drug spending in the United States while accounting for only 2% of all US prescriptions [27].

Neither legislation nor executive action has provided for full patent disclosure in the Purple Book. The Biosimilars Act instituted the “patent dance,” but the exchanges of patent lists in the dance were private; nothing in the Act required public patent disclosure. The Purple Book Continuity Act of 2020 (“Continuity Act”) contained the first statutory requirement that at least some patent information be published in the Purple Book [13]. The Continuity Act’s attempt to require patent disclosure, however, is severely limited. The disclosure requirement is triggered only by the filing of a biosimilar application and the commencement of the “patent dance.” Moreover, the disclosure is limited to the patents disclosed by the brand biologic maker in its initial patent list provided during the “patent dance” [13,28]. Thus, no disclosure is required if no biosimilar application is filed or if, for example, settlement occurs before the “patent dance” starts. Moreover, because no disclosure is required before the first biosimilar application is filed, the Continuity Act places an asymmetrical burden on the first biosimilar: That Act allows second and subsequent biosimilars to “free-ride” on the first biosimilar’s risk-taking and on the patent disclosure resulting from the first biosimilar’s “patent dance.” The first biosimilar, for all its trouble, receives no market exclusivity at all, and subsequent biosimilars may enter the market at their earliest convenience. Finally, the Continuity Act’s disclosure requirement is not retroactive [16].

An executive order issued on July 9, 2021, directed the Secretary of Health and Human Services to make biosimilar approval “more transparent, efficient, and predictable” [29]. The order, however, could do nothing to change the wording of the Continuity Act.

To alleviate the difficulties faced by biosimilars and to reduce the financial burden on patients and insurers, Congress and the FDA must address the informational void that exists in the Purple Book. Congress should first require brand biologics to disclose patent rights in their BLAs—mirroring the disclosures required for brand small-molecule companies—with provisions for supplementary disclosures as needed. The FDA, in turn, should then publish and ensure timely updates of that information in the Purple Book, just as the FDA already does for small-molecule drugs in the Orange Book. Failure to list all patent rights in BLAs would be deterred by a more robust “list it or lose it” provision that bars suits for infringement of patents omitted not only from the brand’s initial patent list but also from the BLA itself. The downside of that enhanced bar—incentivizing disclosure of doubtfully relevant as well as genuinely relevant patents—would be more than outweighed by the informational boon to future biosimilars. Moreover, improper disclosure of patents is anticompetitive and could be readily addressed by the Federal Trade Commission (FTC); indeed, the FTC has recently exercised its authority to bar companies from improperly disclosing patents in the Orange Book [30]. Furthermore, Congress should resolve the “free-rider” problem by ensuring that the first successful biosimilar applicant receives a market exclusivity, during which the FDA would be barred from approving other biosimilar applications related to the reference brand biologic.

Going one step beyond the disclosure required of brand small-molecule drug makers, Congress should also require that the patents to be disclosed by brand biologics in their BLAs must include process patents. Scientists do not yet have the ability to completely identify the function, structure, or composition of biologics, whose molecules are large and exceedingly complex [31,32]. Instead, biologics are identified by the process of making them, and it is extremely difficult, if not impossible, to generate any biosimilar without a precise re-creation of the manufacturing process and cell line used in the production of the relevant biologic [32]. Hence, the oft-repeated saying, “The process is the product” [32,33]. Process patents are accordingly of fundamental importance in the world of biologics and should be disclosed in the brand’s BLA.

Once the Purple Book includes all the patents protecting a brand biologic, prospective biosimilar makers will have full knowledge of the patent obstacles they must overcome. That knowledge will facilitate market entry and spur competition. Increasing competition in the marketplace for biologic drugs can reduce the financial burden on patients, improving access to life-saving drugs and increasing compliance with medical regimens.
